# Utilizing Cerebrospinal Fluid Biomarkers for Accurate Diagnosis of Cognitive Decline in a Tertiary Care Memory Center

**DOI:** 10.1002/alz70856_101653

**Published:** 2025-12-25

**Authors:** Yarovinsky Natalya, Rafi Hadad, Iris Heller, Lilach Kapon, Olga Kogan, Michal Levavi, Merav Shor, Natali Manov, Tali Fisher, Judith Aharon‐Peretz, David Tanne, Rachel Ben‐Hayun

**Affiliations:** ^1^ Technion‐Israel Institute of Technology, Haifa, Israel; ^2^ Rambam Health Care Campus, Haifa, Israel; ^3^ Stroke and Cognition Institute, Rambam Health Care Campus, Haifa, Israel; ^4^ Global Brain Health Institute, san Francisco, CA, USA; ^5^ Immunology Laboratory, Rambam Health Care Campus, Haifa, Israel

## Abstract

**Background:**

An accurate clinico‐biological diagnosis of cognitive decline is crucial for identifying neurodegenerative diseases from disorders with similar clinical presentations and administration of appropriate treatments.

Recent biotechnological advancements have enabled the measurement of protein biomarkers associated with Alzheimer's Disease (AD) pathology and the assessment of paraneoplastic and non‐paraneoplastic antibodies indicative of autoimmune processes.

Since June 2020, our service has conducted comprehensive assessments, incorporating neurological, cognitive evaluations, imaging and CSF biomarker workups.

Our aim was to assess the effectiveness of our approach in achieving an accurate clinic‐biological diagnosis of the underlying etiology of cognitive impairment in individuals evaluated at our center.

**Method:**

Consenting patients presenting with cognitive and/or behavioral deterioration underwent lumbar puncture (LP) for cerebrospinal fluid (CSF) analysis, tailored according to the clinical differential diagnosis. The analysis included cell count, protein, glucose, chloride, cytology, AD biomarkers—total tau, 181‐phosphorylated tau, amyloid‐β42—and panels for paraneoplastic and non‐paraneoplastic antibodies.

**Result:**

A total of 131 patients presenting with cognitive and/or behavioral deterioration (mean age 63.4 ± 9.4 years; 60 females, 45%) underwent LP for CSF evaluation. AD biomarkers were assessed in 120 subjects, with 59 (49%) testing positive. Antibodies associated with autoimmune encephalitis were tested in 92 subjects, revealing positive paraneoplastic antibody profiles in 14 (15%) subjects (8 with biomarker‐confirmed AD and 6 with non‐AD pathology). Two patients with positive both AD‐biomarkers and paraneoplastic antibodies passed away 34 and 77 months after the evaluation, 6 received immune treatment, and 5 were diagnosed with an oncological condition.

**Conclusion:**

Our study highlights the significant value of biomarkers in diagnosing cognitive and behavioral impairments, helping to prevent misdiagnosis or inappropriate labeling of Alzheimer's Disease and guiding the appropriate treatment. We suggest that it is essential to include autoimmune encephalopathy in the differential diagnosis for patients presenting with cognitive decline, as appropriate treatment can be life‐saving.

